# Associação da Composição Corporal com Rigidez Arterial em Longevos

**DOI:** 10.36660/abc.20190774

**Published:** 2021-07-07

**Authors:** Flávia Veríssimo Melo e Silva, Franciellen Bruschi Almonfrey, Cinthia Medice Nishide de Freitas, Flávia Kurebayashi Fonte, Mariana Bellaguarda de Castro Sepulvida, Clineu de Mello Almada-Filho, Maysa Seabra Cendoroglo, Egli Belinazzi Quadrado, Celso Amodeo, Rui Povoa, Roberto Dischinger Miranda

**Affiliations:** 1 Universidade Federal de São Paulo Escola Paulista de Medicina São Paulo SP Brasil Universidade Federal de São Paulo – Escola Paulista de Medicina (Unifesp- EPM), São Paulo, SP - Brasil; 2 Universidade Estadual de Campinas Campinas SP Brasil Universidade Estadual de Campinas (UNICAMP), Campinas, SP - Brasil

**Keywords:** Idoso, Gordura, Rigidez Arterial, Composição Corporal, DEXA

## Abstract

**Fundamento:**

Pouco se conhece sobre a relação entre sarcopenia e hemodinâmica central em idosos longevos.

**Objetivo:**

Estudar a relação da rigidez arterial com a composição corporal em idosos longevos.

**Métodos:**

A composição corporal foi avaliada por meio da absortometria de Raio X de dupla energia (DEXA) e dos parâmetros de circulação central (PCC) obtidos por método oscilométrico não invasivo, com o Mobil-O-Graph 24h PWA Monitor®. Os parâmetros centrais avaliados foram: velocidade da onda de pulso (VOP), *augmentation index* (AIx), índice de amplificação da pressão de pulso (iAPP) e pressão de pulso central (PPc). Estes foram correlacionados com massa magra total (MM) e apendicular (MA), percentual de gordura corporal e índice de Baumgartner (IB). Aceitou-se nível de significância de 5%.

**Resultados:**

Participaram 124 longevos, com idade média de 87,1 anos (DP±4,3 anos), sendo 74,2% mulheres e 57,3% brancos. Houve correlação inversa do AIx com as variáveis MM (r = - 0,391, p < 0,001), MA (r= -0,378, p< 0,001) e IB (r = -0,258, p 0,004). A PPc apresentou associação inversa com MM (r= -0,268, p =0,003), MA (r=-0,288, p= 0,001) e IB (r= -0,265, p = 0,003). Houve relação direta apenas entre AIx e percentual de gordura corporal (r= 0,197, p= 0,029).

**Conclusão:**

Em idosos longevos, o percentual de gordura corporal se associa diretamente com a rigidez arterial e tem associação inversa com a quantidade de MM. Esses achados podem estar associados ao maior risco cardiovascular.

## Introdução

O envelhecimento é um fenômeno global, e a população com 80 anos ou mais (idosos longevos) irá triplicar de 2015 a 2050, sendo esse aumento mais acelerado nos países em desenvolvimento.^1^

Em faixas etárias mais elevadas, há maior prevalência de doenças crônicas. Assim, além da própria idade, somam-se vários fatores de risco que elevam a taxa de eventos cardiovasculares (CV), que continuam sendo a principal causa de morbimortalidade nessa faixa etária.^2 - 4^ A rigidez arterial também contribui para o aumento do risco de eventos CV maiores, tais como infarto agudo do miocárdio (IAM) e acidente vascular encefálico (AVE), independentemente da presença de hipertensão arterial (HA), em adultos e idosos jovens.^5 - 7^ Existem poucos estudos correlacionando a rigidez arterial ao risco CV na população de idosos longevos.^6 , 7^

Outro fator que apresenta correlação com o risco CV é a distribuição e a quantidade de gordura e de massa magra (MM) corporal. A gordura visceral abdominal está associada ao aumento da síndrome metabólica e do risco CV, mesmo naqueles que possuem um peso adequado para a altura.^8 , 9^ Esta representa uma melhor associação com o risco CV do que o índice de massa corporal (IMC) na população idosa.^10 - 12^ Além disso, a redução da MM associa-se com maior mortalidade global e CV.^13 , 14^

Alguns estudos sugerem que a rigidez arterial contribua para a relação entre a composição corporal e o aumento do risco CV.^15 - 17^ Entretanto, essa relação não está clara, principalmente quando se trata de idosos longevos, pois apesar de serem uma população em crescimento exponencial, ainda são pouco estudados.

Dessa maneira, mesmo não se conhecendo exatamente o mecanismo que une a obesidade e a sarcopenia ao dano vascular, acredita-se que possa haver associação com a rigidez arterial.^18 , 19^

O objetivo deste trabalho foi avaliar a relação entre rigidez arterial e composição corporal em idosos longevos que vivem na comunidade.

## Método

Foi realizada uma análise transversal de uma coorte prospectiva de idosos com 80 anos ou mais, com independência funcional e cognitiva (Estudo Longevos). Este foi composto tanto por uma avaliação clínica, cognitiva, funcional e nutricional quanto pela realização de exames de rotina e de interesse científico. Todos os participantes assinaram um termo de consentimento livre e esclarecido para serem admitidos no programa.

Foram excluídos os indivíduos que apresentavam: insuficiência renal em programa de diálise; instabilidade hemodinâmica com necessidade de uso de fármacos vasoativos; insuficiência cardíaca com classe funcional III ou IV; doenças psiquiátricas que pudessem comprometer a realização das avaliações propostas no protocolo; doenças graves e/ou câncer com prognóstico menor que um ano.

Esta análise inclui todos aqueles indivíduos que realizaram uma avaliação da composição corporal e uma medida não invasiva dos parâmetros da circulação central (PCC), com um intervalo de tempo de no máximo um ano entre elas. Projeto aprovado pelo Comitê de Ética em Pesquisa da Universidade Federal de São Paulo (CEP- UNIFESP) com Nº CEP 0190/2017 e Nº do parecer 2.381.489.

Os PCC foram obtidos de modo não invasivo com a utilização do equipamento Mobil-O-Graph 24h PWA Monitor® (I.E.M. GmbH, Stolberg, Germany) validado para essa finalidade, o qual capta simultaneamente a pressão arterial braquial e a forma da onda de pulso.^20 - 22^

A pressão arterial foi aferida em todos os pacientes no dia de aplicação do protocolo, usando como base as recomendações da VII Diretriz Brasileira de Hipertensão Arterial.^23^ Utilizamos a média das medidas válidas dos seguintes parâmetros centrais: velocidade da onda de pulso (VOP), *augmentation index* (Alx), índice de amplificação da pressão de pulso (iAPP), pressão de pulso central (PPc), e os correlacionamos aos dados da composição corporal.

A composição corporal foi avaliada pela DEXA (Absortometria de Raio X de Dupla Energia ou Dual Energy X-Ray Absorptiormetry) com o equipamento de densitometria Hologic (Modelo Discovery A, Waltham, USA), permitindo quantificar a MM total, a massa apendicular (MA) e o percentual de gordura corporal.^24 , 25^ A MA foi calculada pela razão da quantidade de MM em braços e pernas, em gramas, e a altura do indivíduo ao quadrado, em metros (MA em g/m^2^). Além disso, a DEXA permite calcular o índice de Baumgartner (IB), que utiliza a MA no numerador da fórmula do IMC, em substituição à massa corporal total.

### Análise estatística

Como o estudo utilizou uma coorte prospectiva de idosos longevos, o tamanho da amostra foi decidido por conveniência. Inicialmente os dados foram analisados descritivamente.

As variáveis categóricas foram apresentadas como frequências absolutas e relativas. As variáveis contínuas numéricas com distribuição normal foram descritas como média e desvio-padrão.

Para se avaliar a associação linear entre duas variáveis numéricas, foi utilizada a correlação de Pearson. Foram apresentadas também as correlações parciais ajustadas pela idade.

Para todos os testes estatísticos, foi utilizado um nível de significância de 5%.

As análises estatísticas foram realizadas com o uso do software estatístico SPSS 20.0.

## Resultados

Foram analisados 124 idosos longevos, dos 74,2% eram mulheres e 57,3% eram de cor branca, com média de idade de 87,1±4,3 anos, sendo observada uma idade mínima de 80 anos e máxima de 100 anos.

O tempo médio entre a realização das medidas de pressão e da DEXA foi 43,4 dias. Da amostra obtida, 41,9% eram eutróficos, pela classificação de Lipschitz (IMC 22 a 27 kg/m^2^) e 41,1% obesos (IMC>27 kg/m^2^). Na distribuição do percentual de gordura obtido na DEXA em tercis, percebeu-se que 1/3 da amostra possuía mais de 42,9% da composição corporal composta por massa gorda, 1/3 possuía de 36,4 a 42,9%, e 1/3 possuía menos de 36,4% de massa gorda.

**Figura 1:**
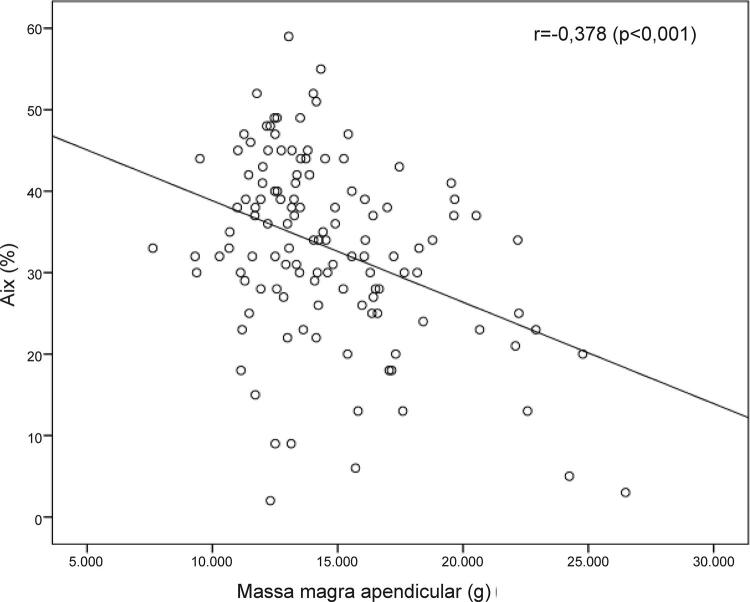
– *Gráfico de dispersão mostrando a relação inversa (r= 0,378; p<0,001) entre AIx (%) e massa magra apendicular (g).*

Houve uma associação inversa da quantidade de MM, seja apendicular ou total, com a rigidez arterial ( Figura 1 ). Observou-se associação estatisticamente significante de dois PCC (PPc e AIx) com a MM ( Tabela 1 ).

**Tabela 1:** – Correlação de Pearson entre as variáveis de rigidez arterial e a massa magra corporal, após ajuste pela idade

	MM (g)		Massa apendicular (g)		Indice de Baumgartner (Kg/m^2^)	

	r	p	r	p	r	p
PPc (mmHg)	-0,267*	0,003	-0,283*	0,002	-0,263*	0,003
AIx (%)	-0,393**	<0,001	-0,382**	<0,001	-0,260**	<0,004
VOP (m/s)	-0,052	0,571	-0,082	0,369	-0,102	0,263
iAPP (%)	0,168	0,063	0,147	0,106	0,128	0,159

* p<0,01; ** p<0,001. N=124; MM (massa magra total); PPc (pressão de pulso central); AIx (augmentation index); VOP (velocidade de onda de pulso): iAPP (índice de ampliação da pressão de pulso).

Foi observada uma associação inversamente proporcional entre o AIx e as variáveis MM (r=-0,391, p<0,001), MA (r=-0,378, p<0,001) e IB (r=-0,258, p=0,004). A PPc também apresentou significância estatística em suas relações inversas com a MM (r = -0,268, p = 0,030), MA (r= -0,288, p = 0,001) e IB (r = -0,265, p = 0,003). A VOP não apresentou significância estatística nesta análise.

Houve relação direta, mas sem significância estatística, entre iAPP e MM ( Tabela 1 ).

As correlações entre as variáveis de rigidez arterial e composição corporal foram ajustadas pela idade, e os resultados apresentaram-se semelhantes ( Tabela 1 e Figura 1 ). Assim, o AIx continuou a apresentar relação inversa com a quantidade de MM e o IB. O mesmo ocorreu na comparação ajustada pela idade entre a PPc e a MM. ( Tabela 1 )

Ao avaliar a relação da gordura corporal e os parâmetros da rigidez arterial, houve uma relação direta e estatisticamente significante entre o AIx e o percentual de gordura corporal, mas não com o peso total de gordura corporal (r= -0,029, p= 0,746). Esta associação se manteve após ajuste para a idade, conforme demonstrado na Tabela 2 . Os demais parâmetros analisados (VOP, iAPP e PPc) não apresentaram relação estatística com a gordura corporal. Nenhum dos parâmetros de rigidez arterial analisados (PPc, AIx, VOP, iAPP) mostrou relação com o IMC ( Tabela 2 ).

**Tabela 2:** – Correlação de Pearson entre as variáveis de rigidez arterial e gordura corporal após o ajuste para a idade

	MM (g)		% massa gorda	

	r	p	r	p
PPc (mmHg)	-0,004	0,962	0,174	0,054
AIx (%)	-0,042	0,642	0,197*	0,029
VOP (m/s)	0,005	0,952	0,048	0,601
iAPP (%)	0,064	0,479	-0,042	0,647

* p<0,05. N=124; MG (massa gorda); PPc (pressão de pulso central); AIx (augmentation index); VOP (velocidade de onda de pulso); iAPP (índice de ampliação da pressão de pulso)

## Discussão

Este é o primeiro estudo a avaliar a associação entre a rigidez arterial e composição corporal em idosos longevos. Nesta avaliação, houve relação entre a composição corporal e rigidez arterial, sendo que, quanto maior a quantidade de massa muscular, menor a rigidez da parede das artérias centrais. Esse resultado se deu pela associação do AIx e da PPc com a MM e a MA, com significância estatística (p<0,001), mesmo após a correção para a idade.

Observou-se também relação direta entre o percentual de gordura corporal e a rigidez arterial por meio do AIx (p= 0,029).

Esses resultados foram equivalentes àqueles encontrados em alguns estudos com adultos de meia idade. Wohlfahrt et al. avaliaram 136 voluntários com idade média de 45 anos, não obesos e sem doenças CV.^19^ Concluíram que os indivíduos com maior MM e menor percentual de gordura possuíam artérias mais elásticas, e também foi mais forte a associação da rigidez aórtica com a MM do que com a massa gorda. Schouten et al.,^26^ acompanharam adultos saudáveis e constataram que o aumento da gordura abdominal ou a perda de MA estava relacionado à maior rigidez arterial.^26^

Por outro lado, Benetos et al.,^27^ ao analisarem 169 idosos, não encontraram associação da MM com a VOP, apenas com a gordura corporal. Porém, a amostra desse estudo era formada apenas por homens e com uma faixa etária mais jovem de 60 a 85 anos, em que 90% da amostra tinha abaixo de 75 anos. Além disso, não foram avaliados outros marcadores de rigidez arterial, como a PPc e o AIx.

Já Tanaka et al.,^28^ avaliaram uma população exclusivamente de mulheres diabéticas, com idade média de 65 anos, e apontaram a associação de rigidez arterial, medida pela VOP, tanto com o percentual de gordura corporal quanto com a MM.^28^

No presente estudo, não houve relação estatística entre VOP e composição corporal, diferentemente dos artigos supracitados. Porém, o estudo PARTAGE (Predictive Values of Blood Pressure and Arterial Stiffness in Institutionalized Very Aged Population), realizado com 1.126 idosos com mais de 80 anos, ou seja, com idade similar à dos participantes do presente estudo, também não demonstrou correlação da VOP com desfechos CV e mortalidade global.^7^ Nesse estudo, o iAPP se mostrou o melhor marcador de risco para os eventos, podendo sugerir que, em idosos longevos, para medir a rigidez arterial, além da VOP, outros parâmetros devam ser analisados. É necessário destacar que o estudo aqui apresentado avaliou idosos da comunidade, enquanto, no PARTAGE, os idosos eram institucionalizados, apesar de também independentes para atividades básicas de vida diária.

Para a obtenção dos parâmetros arteriais centrais, utilizamos o equipamento não invasivo Mobil-O-Graph 24h PWA Monitor®, que utiliza um método oscilométrico validado. Uma revisão sistemática com metanálise publicada recentemente comparou os equipamentos comercialmente existentes para aferição indireta dos PCC.^29^ Sua conclusão foi de que não há evidências suficientes para recomendar uma forma não invasiva de estimar a pressão arterial central como padrão-ouro. Apesar de a tecnologia do SphygmoCor® ter sido a mais estudada e validada até então, apresenta maior erro na estimativa da pressão arterial na raiz da aorta em relação ao método invasivo, o que dispositivos que usam um método automático oscilométrico. Estes possuem maior acurácia e parecem ser mais promissores.

No vigente estudo, foi utilizada a ferramenta de escolha para avalição da densidade mineral óssea conforme recomendação do Consenso Europeu de Sarcopenia para avaliação da MA na prática clínica, mas não há consenso sobre seu uso para a definição de obesidade.^30 , 31^

A fraca associação encontrada entre rigidez arterial e gordura corporal corrobora a teoria do paradoxo da obesidade no idoso.^32^ A menor associação de doenças CV com a obesidade pode ocorrer devido um viés de sobrevivência, em que os idosos que chegaram a essa faixa etária não estão suscetíveis aos malefícios metabólicos do excesso de gordura. Contudo, é importante enfatizar que, apesar de não possuir correlação com a mortalidade após os 75 anos, o excesso de gordura corporal aumenta a chance de fragilidade e de perda da funcionalidade, levando à dependência para atividades de vida diária.^33^

Dentre as limitações do presente estudo, podemos destacar que foi conduzido em centro único, em população de muitos idosos da comunidade, não permitindo generalização dos seus resultados para populações diversas. O tamanho amostral, relativamente pequeno, pode ter sido um importante limitador para os achados. Porém, este é o primeiro estudo específico para a população de 80 anos ou mais que vive na comunidade e não está institucionalizada, cujo acesso para pesquisa clínica é mais limitado, devido às dificuldades inerentes ao envelhecimento.

O desenho deste estudo não permite sugerir um mecanismo ou uma relação de causa e efeito entre as associações encontradas. Não foram levadas em consideração outras variáveis que podem interferir no enrijecimento vascular, tais como: hipertensão, diabetes, síndrome metabólica e dislipidemia. Também não foram avaliadas as medicações em uso, como os anti-hipertensivos, que podem influenciar diretamente os PCC.^34^

Estudos longitudinais de coorte e ensaios clínicos são necessários para a confirmação das relações apresentadas com os desfechos CV,

## Conclusão

O percentual de gordura corporal se associa diretamente com a rigidez arterial, em idosos longevos, enquanto a quantidade de MM tem associação inversa.

Levando-se em conta que a rigidez arterial pode ter relação direta com o aumento do risco CV, podemos levantar a hipótese de que a massa muscular esteja inversamente associada com o risco de desfechos CV em idosos longevos.
