# Supplementary material for: Associação da Composição Corporal com Rigidez Arterial em Longevos
Source: Arq Bras Cardiol. 2021 Jul 7;117(3):457–62. [Article in Portuguese] doi: 10.36660/abc.20190774 (PMC8462957; doi:10.36660/abc.20190774)
Supplement: Translation

# Translation: Association of Body Composition with Arterial Stiffness in Long-lived People

**Authors:** Flávia Veríssimo Melo e Silva, Franciellen Bruschi Almonfrey, Cinthia Medice Nishide de Freitas, Flávia Kurebayashi Fonte, Mariana Bellaguarda de Castro Sepulvida, Clineu de Mello Almada-Filho, Maysa Seabra Cendoroglo, Egli Belinazzi Quadrado, Celso Amodeo, Rui Povoa, Roberto Dischinger Miranda

**Affiliations:** 1 Brasil Universidade Federal de São Paulo – Escola Paulista de Medicina (Unifesp- EPM), São Paulo, SP - Brasil; 2 Brasil Universidade Estadual de Campinas (UNICAMP), Campinas, SP - Brasil

**Keywords:** Aged, Fats, Vascular Stiffness, Body Composition, DEXA

## Abstract

**Background:**

Arterial stiffness, obesity and sarcopenia correlate with each other and with cardiac outcomes in younger adults. However, there is little evidence of the association between body composition and markers of central arteries stiffness in long-lived people.

**Objective:**

To evaluate the relationship between arterial stiffness and body composition in functionally independent long-lived individuals.

**Methods:**

This is a cross-sectional analysis of the association between markers of arterial stiffness and body composition among participants in a longitudinal cohort of elderly individuals aged 80 years or older who were functionally independent and lived in the community . Body composition measurements were performed using dual energy X-ray absorptiometry (DEXA) and central circulation parameters (CCP) obtained by a non-invasive oscillometric method through the Mobil-O-Graph 24h PWA Monitor® device. The central parameters evaluated were: pulse wave velocity (PWV), augmentation Index (AIx), pulse pressure amplification index (PPAi) and central pulse pressure (cPP). These were correlated to total lean mass (LM) and appendicular lean mass (aLM), body fat percentage, and Baumgartner’s Index (BI). The level of significance was set at 5% for all tests.

**Results:**

Data from 124 elderly people with a mean age of 87.1 years (SD ± 4.3 years) were analyzed, with 74.2% of women and 57.3% of white. There was a statistically significant inverse correlation of AIx with LM (r = -0.391, p <0.001), aLM (r = -0.378, p <0.001), and BI (r = -0.258, p = 0.004). Also, cPP had an inversely proportional association with LM (r = -0.268, p = 0.003), aLM (r = -0.288, p = 0.001), and BI (r = -0.265, p = 0.003). When assessing the relationship between fat mass and CCP, a statistically significant direct relationship was observed only between AIx and body fat percentage (r = 0.197, p = 0.029).

**Conclusion:**

In long-lived people, body fat percentage is directly associated with arterial stiffness and inversely associated with the amount of LM. These findings may be associated with increased cardiovascular risk.

## Introduction

Aging is a worldwide phenomenon, and the population aged 80 years or older (long-lived people) will experience a threefold increase from 2015 to 2050, with a more accelerated increase in developing countries.^1^

The prevalence of chronic diseases is higher at older ages. Thus, in addition to age itself, several risk factors increase the rate of cardiovascular (CV) events, which is still the main cause of morbidity and mortality in this age group.^2 - 4^ Arterial stiffness also contributes to increase the risk of major CV events, such as acute myocardial infarction (AMI) and stroke, regardless of the presence of arterial hypertension (AH), in adults and younger elderly people.^5 - 7^ There are few studies correlating arterial stiffness with CV risk among long-lived people.^6 , 7^

Another factor correlated with CV risk is the distribution and amount of fat and body lean mass. Abdominal visceral fat is associated with increased metabolic syndrome and with CV risk, even in those with adequate weight for their height.^8 , 9^ This type of fat has a better association with CV risk than body mass index (BMI) in the elderly population.^10 - 12^ Furthermore, reduction in lean mass is associated with greater overall and CV mortality.^13 , 14^

Some studies suggest that arterial stiffness contributes to the relationship between body composition and increased CV risk.^15 - 17^ However, this relationship is not clear, especially with regard to long-lived people, because, although this population has been experiencing exponential growth, they have been little studied.

Therefore, even though the mechanism linking obesity and sarcopenia with vascular damage is not well known, it is believed that there may be an association with arterial stiffness.^18 , 19^

The aim of this study was to assess the relationship between arterial stiffness and body composition in long-lived people from the community.

## Methods

A cross-sectional analysis was conducted with a prospective cohort of older adults aged 80 years or older and with functional and cognitive independence (Estudo Longevos). This study consisted of both clinical, cognitive, functional, and nutritional assessments and routine tests of scientific interest. All participants signed a free informed consent to being included in the program.

The study excluded individuals with renal failure undergoing dialysis; with hemodynamic instability requiring vasoactive drug therapy; with functional class III or IV heart failure; with psychiatric diseases that could compromise the execution of the assessments proposed in the protocol; and with severe diseases and/or cancer with life prognosis of less than a year.

This analysis comprised all individuals who underwent body composition assessment and non-invasive measurement of central circulation parameters (CCP), with an interval of no more than a year between them. The study project was approved by the Research Ethics Committee of Universidade Federal de São Paulo (CEP-UNIFESP) under CEP no. 0190/2017 and opinion no. 2.381.489.

CCP were non-invasively obtained using the Mobil-O-Graph 24h PWA Monitor® device (I.E.M. GmbH, Stolberg, Germany) validated to this end, which simultaneously measures brachial arterial pressure and pulsewave form.^20 - 22^

Blood pressure was taken in all patients on the day of protocol implementation, based on the recommendations of the VII Brazilian Guidelines on Arterial Hypertension.^23^ Mean for the valid measurements of the following central parameters were used: pulse wave velocity (PWV), augmentation index (AIx), pulse pressure amplification index (PPAi), central pulse pressure (cPP), and correlated them with data on body composition.

Body composition was assessed by DEXA (Dual Energy X-Ray Absorptiormetry) using a Hologic bone densitometry machine (Model Discovery A, Waltham, USA), which allowed to quantify total lean mass (LM), appendicular lean mass (aLM), and body fat percentage.^24 , 25^ aLM was calculated by the ratio between the amount of lean mass in arms and legs, expressed in grams, and the squared height in meters (aLM in g/m^2^). Moreover, DEXA allows calculating the Baumgartner index (BI), which uses aLM in the numerator of the BMI formula instead of total body mass.

### Statistical analysis

Since the study used a prospective cohort of very old adults, sample size was chosen by convenience. Initially, data were descriptively analyzed.

Categorical variables were expressed as absolute and relative frequencies. Numerical continuous variables with normal distribution were described as mean and standard deviation.

The linear association between two numerical variables was assessed using the Pearson’s correlation test. Partial correlations adjusted for age were also presented.

The level of significance for all statistical tests was set at 5%.

Statistical analyses were performed using the SPSS 20.0 statistical software.

## Results

A total of 124 long-lived people were assessed, of which 74.2% were women and 57.3% were white, with a mean age of 87.1±4.3 years, minimum of 80 years and maximum of 100 years.

Mean time elapsed from blood pressure measurements and DEXA assessments was 43.4 days. Of the total participants, 41.9% had normal weight, according to the Lipschitz classification (BMI from 22 to 27 kg/m^2^) and 41.1% were obese (BMI>27 kg/m^2^). With regard to the distribution of body fat percentage as assessed by DEXA and divided into tertiles, 1/3 of the sample was shown to have more than 42.9% fat mass in their body composition, whereas 1/3 had from 36.4 to 42.9% of fat mass and 1/3 had less than 36.4% of fat mass.

There was an inverse association between lean mass, either appendicular or total, and arterial stiffness ( Figure 1 ). A statistically significant association was observed between two CCPs (cPP and AI) and lean mass ( Table 1 ).

**Figure 1:** – *Dispersion graph showing the inverse relationship (r= 0.378; p<0.001) between AI (%) and appendicular lean mass (g).*

**Table 1:** – Pearson’s correlation between variables of arterial stiffness and body lean mass after adjustment for age

	LM (g)		Appendicular lean mass (g)		Baumgartner index (kg/m^2^)	

	r	p	r	p	r	p
cPP (mmHg)	-0.267*	0.003	-0.283*	0.002	-0.263*	0.003
AI (%)	-0.393**	<0.001	-0.382**	<0.001	-0.260**	<0.004
PWV (m/s)	-0.052	0.571	-0.082	0.369	-0.102	0.263
PPAi (%)	0.168	0.063	0.147	0.106	0.128	0.159

* p<0.01; ** p<0.001. N=124 LM (total lean mass); cPP (central pulse pressure); AI (augmentation index); PWV (pulse wave velocity): PPAi (pulse pressure amplification index)

An inversely proportion association was found between AI and the variables LM (r=-0.391, p<0.001), aLM (r=-0.378, p<0.001) and BI (r=-0.258, p=0.004). There was also a statistically significant inverse relationship between cPP and LM (r = -0.268, p = 0.030), aLM (r= -0.288, p = 0.001), and BI (r = -0.265, p = 0.003). PWV did not show any statistical significant association in this analysis.

A direct relationship, but with no statistical significance, was found between PPAi and LM ( Table 1 ).

These correlations between variables of arterial stiffness and body composition were adjusted for age, yielding similar results ( Table 1 and Figure 1 ). Thus, AI still had an inverse relationship with LM and IB. This also occurred in the age-adjusted comparison between cPP and LM. ( Table 1 )

An assessment of relationship between body fat and parameters of arterial stiffness showed a statistically significant direct relationship between AI and body fat percentage, but not between AI and total body fat (r= -0.029, p= 0.746). This association remained after adjustment for age, as shown in Table 2 . The other analyzed parameters (PWV, PPAi and cPP) did not have a statistical relationship with body fat. None of the analyzed parameters of arterial stiffness (cPP, AI, PWV, PPAi) exhibited a relationship with BMI ( Table 2 ).

**Table 2:** – Pearson’s correlation between variables of arterial stiffness and body fat after adjustment for age

	FM (g)		Body fat percentage	

	r	p	r	p
cPP (mmHg)	-0.004	0.962	0.174	0.054
AI (%)	-0.042	0.642	0.197*	0.029
PWV (m/s)	0.005	0.952	0.048	0.601
PPAi (%)	0.064	0.479	-0.042	0.647

* p<0.05; N=124; FM (fat mass); cPP (central pulse pressure); AI (augmentation index); PWV (pulse wave velocity); PPAi (pulse pressure amplification index)

## Discussion

This is the first study to assess the association between arterial stiffness and body composition in long-lived people. This assessment found a relationship between body composition and arterial stiffness, showing that the greater the amount of muscle mass, the lower the wall stiffness of central arteries. This result may be explained by the association of AI and cPP with total and aLM, with statistical significance (p<0.001), even after correction for age.

A direct relationship was observed between body fat percentage and arterial stiffness as assessed by AI (p= 0.029).

These results were consistent with those found in some studies with middle-aged adults. Wohlfahrt et al. assessed 136 volunteers with mean age of 45 years, non-obese, and without CV diseases.^19^ They concluded that individuals with greater LM and lower fat percentage had more elastic arteries and also observed a stronger association between aortic stiffness and LM compared with fat mass. Schouten et al.^26^ followed healthy adults and found that increased abdominal fat or loss of aLM was related to greater arterial stiffness.^26^

Conversely, Benetos et al.,^27^ when analyzing 169 older adults, found that LM associated with body fat, but not with PWV. However, the sample of this study only included men of a younger age (60 to 85 years), of which 90% were aged below 75. Furthermore, other markers of arterial stiffness, such as cPP and AI, were not assessed.

Tanaka et al.,^28^ in turn, evaluated a population consisting exclusively of diabetic women with mean age of 65 years and showed the association of arterial stiffness, as measured by PWV, both with body fat percentage and with LM.^28^

In the present study, there was no statistical relationship between PWV and body composition, contrary to above mentioned articles. However, the PARTAGE (Predictive Values of Blood Pressure and Arterial Stiffness in Institutionalized Very Aged Population) study, conducted with 1,126 elderly individuals aged 80 years or older, i.e., with similar age than that of participants of the present study, did not observe a correlation between PWV and CV outcomes and overall mortality.^7^ In the PARTAGE study, PPAi showed to be the best risk marker for these events, which may suggest that other parameters should be analyzed to measure arterial stiffness among long-lived people, in addition to PWV. It is necessary to highlight that the study presented here assessed older adults from the community, whereas the PARTAGE study assessed older adults who were institutionalized, although also independent for basic activities of daily living.

Central arterial parameters were measured using Mobil-O-Graph 24h PWA Monitor®, a non-invasive equipment that uses a validated oscillometric method. A recently published systemic review with meta-analysis compared the commercially available equipment for indirect measurement of CCP.^29^ It concluded that there is no sufficient evidence to recommend a non-invasive technique to estimate a central blood pressure as a gold-standard. Although the technology of SphygmoCor® has been more studied and validated so far, it shows a greater number of errors in blood pressure estimation on the aortic root with regard to the invasive method, more than devices that use an automatic oscillometric method. These devices have greater accuracy and seem more promising.

The present study used the tool of choice for bone mineral density as recommended by the European Consensus on Sarcopenia for the assessment of appendicular muscle mass in clinical practice, but there was no consensus on its use to define obesity.^30 , 31^

The weak association between arterial stiffness and body fat found in this study corroborates the theory of the obesity in the elderly.^32^ The lower association between CV diseases and obesity may be explained by a survival bias, in which individuals who reach old age are not susceptible to the metabolic hazards of excess fat. However, it is important to emphasize that, although there was excess body fat is not correlated with mortality after the age of 75, it increases the likelihood of frailty and loss of functionality, leading to dependency for activities of daily living.^33^

One of the remarkable limitations of the present study is the fact that it was conducted in a single center, with a population of many older adults from the community, which does not allow for the generalization of its results to diverse populations. Moreover, the relatively small sample size may be an important limiting factor for its findings. However, this was the first study to specifically assess the population aged 80 years or older living in the community and not institutionalized, whose access to clinical research is more limited, due to difficulties inherent to aging.

The design of this study does not allow suggesting a mechanism or a cause and effect relationship between the found associations. Other variables that may interfere with vascular hardening were not considered, such as: hypertension, diabetes, metabolic syndrome, and dyslipidemia. Furthermore, the present study did not assess medications currently used, such as anti-hypertensive, which could have a direct influence on CCP.^34^

Cohort longitudinal studies and clinical trials are needed to confirm the relationships presented with CV outcomes.

## Conclusion

In long-lived people, body fat percentage is directly associated with arterial stiffness and inversely associated with the amount of LM.

Considering that arterial stiffness may have a directly relationship with increased CV risk may lead to the hypothesis that muscle mass is inversely associated with risk of CV outcomes in long-lived people.
